# MLIF Alleviates SH-SY5Y Neuroblastoma Injury Induced by Oxygen-Glucose Deprivation by Targeting Eukaryotic Translation Elongation Factor 1A2

**DOI:** 10.1371/journal.pone.0149965

**Published:** 2016-02-26

**Authors:** Qiuzhen Zhu, Yuefan Zhang, Yulan Liu, Hao Cheng, Jing Wang, Yue Zhang, Yaocheng Rui, Tiejun Li

**Affiliations:** 1 Department of Pharmacology, College of Pharmacy, Second Military Medical University, Shanghai, China; 2 College of Pharmacy, Anhui University of Chinese Medicine, Hefei, Anhui, China; University of Pecs Medical School, HUNGARY

## Abstract

Monocyte locomotion inhibitory factor (MLIF), a heat-stable pentapeptide, has been shown to exert potent anti-inflammatory effects in ischemic brain injury. In this study, we investigated the neuroprotective action of MLIF against oxygen-glucose deprivation (OGD)-induced injury in human neuroblastoma SH-SY5Y cells. MTT assay was used to assess cell viability, and flow cytometry assay and Hoechst staining were used to evaluate apoptosis. LDH assay was used to exam necrosis. The release of inflammatory cytokines was detected by ELISA. Levels of the apoptosis associated proteins were measured by western blot analysis. To identify the protein target of MLIF, pull-down assay and mass spectrometry were performed. We observed that MLIF enhanced cell survival and inhibited apoptosis and necrosis by inhibiting p-JNK, p53, c-caspase9 and c-caspase3 expression. In the microglia, OGD-induced secretion of inflammatory cytokines was markedly reduced in the presence of MLIF. Furthermore, we found that eukaryotic translation elongation factor 1A2 (eEF1A2) is a downstream target of MLIF. Knockdown eEF1A2 using short interfering RNA (siRNA) almost completely abrogated the anti-apoptotic effect of MLIF in SH-SY5Y cells subjected to OGD, with an associated decrease in cell survival and an increase in expression of p-JNK and p53. These results indicate that MLIF ameliorates OGD-induced SH-SY5Y neuroblastoma injury by inhibiting the p-JNK/p53 apoptotic signaling pathway via eEF1A2. Our findings suggest that eEF1A2 may be a new therapeutic target for ischemic brain injury.

## Introduction

Stroke is the second leading cause of death, and the number of new stroke cases continues to rise along with an ageing population [1]. Ischemic stroke, which accounts for 80% of all strokes, is a devastating disorder with a complex pathophysiology involving inflammation, apoptosis, excitotoxicity, and oxidative and nitrosative stress to brain tissue [2]. The main drug treatment for ischemic stroke is tissue plasminogen activator, which has a very limited time window of therapeutic efficacy. Consequently, there is an urgent clinical need for effective anti-ischemic cerebroprotective drugs.

Monocyte locomotion inhibitory factor (MLIF,Met-Gln-Cys-Asn-Ser), is a heat-stable anti-inflammatory oligopeptide derived from *Entamoeba histolytica* axenic cultures. MLIF has been shown to have numerous biological effects. It is involved in the inflammatory response and in the repair process, it regulates the expression of immunomodulatory genes, and it plays a role in cell proliferation, extracellular matrix production and degradation, vasculogenesis, axon guidance and cellular movement [3, 4]. We previously found that MLIF attenuates the inflammatory response and oxidative damage in focal ischemia and protects cerebrovascular endothelial cells following hypoxic injury by inhibiting the expression of adhesion molecules and by targeting the eEF1A1/eNOS pathway [5]. Yao and colleagues also reported that MLIF is neuroprotective against cerebral ischemia [6].

Eukaryotic translation elongation factor 1 alpha (eEF1A), a member of the G protein family, transfers aminoacylated-tRNAs (aa-tRNAs) to the A site of the ribosome during the elongation cycle in protein biosynthesis [7]. Recently, studies have shown that in addition to the role in protein translation, the two sister genes, eEF1A1 and eEF1A2, exhibit some non-canonical functions. eEF1A1 has been extensively studied [8–11], but eEF1A2 has not. eEF1A2, unlike eEF1A1 which is widely expressed, is mainly expressed in the brain, heart and skeletal muscle [12, 13]. eEF1A1 is gradually replaced by eEF1A2 after birth in the developing brain, and as a result eEF1A2 is the main form in the mature brain [14]. An increasing number of studies have shown that eEF1A2, in addition to its anti-apoptotic properties in many cancers [15–17], has an important role in nervous system diseases. Deletion of the eEF1A2 gene results in a neurodegenerative phenotype [18–21].

MLIF appears to provide neurovascular protection in brain ischemia by modulating the expression of inflammatory adhesion molecules and by regulating endothelial nitric oxide synthase and nitric oxide levels via the eEF1A1/eNOS pathway [5]. However, the mechanisms underlying the neuroprotective actions of MLIF in ischemic brain injury remain unclear.

In the present study, we used an *in vitro* model of ischemia using primary neurons and human neuroblastoma SH-SY5Y cells to study the neuroprotective effects of MLIF. To evaluate the cytoprotective actions of MLIF, cell viability and apoptosis were evaluated in SH-SY5Y cells. In addition, molecular targets of MLIF were identified using pull-down assay and mass spectrometry in SH-SY5Y cells. Furthermore, we used RNAi to clarify the mechanisms underlying the neuroprotective effects of MLIF against OGD-induced SH-SY5Y cells injury.

## Materials and Methods

### Reagents

MLIF and biotinylated MLIF were synthesized by the Chinese Peptide Company (Hangzhou, China), with purity above 98%. MLIF was dissolved in PBS (pH7.4) to a final concentration of 4 mg/ml and stored at −80°C. Rabbit monoclonal antibody specific for eEF1A2 was purchased from Abcam (Cambridge, MA, USA), p-JNK, caspase3, caspase9, ERK, p-ERK, p38, p-p38 and p53 antibodies were purchased from Cell Signaling Technology (Danvers, MA, USA). Secondary antibody was purchased from Kangchen (Shanghai, China), and anti-rabbit phycoerythrin antibody was purchased from Santa Cruz Biotechnology (Santa Cruz, CA). 2-(4-Amidinophenyl)-6-indolecarbamidine dihydrochloride (DAPI) and Hoechst 33258 were obtained from Sigma-Aldrich Co (St. Louis, MO, USA), while 3-(4,5-Dimethylthiazol-2-yl)-2,5-diphenyltetrazolium bromide (MTT) was purchased from Shanghai Chemical Reagent Company (Shanghai, China). All reagents in this study were of analytical grade.

### Cell culture

Human neuroblastoma SH-SY5Y cells and mouse microglial BV-2 cells were purchased from the Cell Bank of Chinese Academy of Sciences (Shanghai, China) and cultured in Dulbecco’s modified Eagle’s medium (Hyclone, Logan, UT, USA) supplemented with 10% heat-inactivated fetal bovine serum (Gibco, Carlsbad, CA, USA), and 1×Penicillin-Streptomycin (100U/ml or 100mg/ml) (Thermo Scientific, Waltham, MA, USA) in a humidified incubator with 5% CO_2_ and 95% air at 37°C. Cells in the log growth phase were used for the various experiments. Rat primary cortical neurons were prepared as described previously [22,23]. Primary neurons were cultured in Neurobasal medium (Life Technologies) supplemented with 2% B27 (Life Technologies) and 0.5 mM L-glutamine, and 1×Penicillin-Streptomycin. Cells were plated at 1x10^6^ cells/ml in polyethyleneimine-coated 96-well plates.

### Oxygen-glucose deprivation and reperfusion procedure

*In vitro* ischemic injury was induced by oxygen-glucose deprivation, as described previously [24]. Cells were cultured until 80% confluent and incubated with glucose-free DMEM in a modular hypoxia incubation chamber (Billups-Rothenberg, Del Mar, CA, USA) with 95% N_2_ and 5% CO_2_ [25]. The chamber was kept in an incubator for 6 hours at 37°C to produce OGD. OGD/R is composed of oxygen-glucose deprivation (OGD) and reoxygenation period. Cells were kept in the glucose-free DMEM for 4 hours in the hypoxia incubation chamber at 37°C. OGD was terminated by adding glucose to a final concentration of 4.5mg/ml followed by incubation in a normoxic incubator for 12 hours (reoxygenation). Control cultures were maintained in an incubator under atmospheric oxygen levels and normal humidity (normoxia, control). A stock solution of MLIF was diluted with cell culture medium just prior to the experiments and added to the cells 2 h before OGD and at the reoxygenation.

### MTT assay

Cells were seeded at 5 × 10^4^ cells/ml and subjected to OGD as described above. After OGD, MTT (20 μL, 5 mg/ml) was added to the cells and incubated at 37°C for 4 h. A 150 μL aliquot of dimethyl sulfoxide was added and incubated for 10 min to dissolve the dark blue crystals. The absorbance was subsequently measured at 490 nm on a microplate reader (Multiskan MK3, Thermo Scientific, USA). The control group without OGD treatment was taken as 100% cell survival and all other groups were normalized to this value.

### Flow cytometry

Cells were seeded at 1.5 × 10^5^ cells/ml, and OGD was performed. The number of apoptotic cells was assessed using an annexin V-FITC/PI assay according to the manufacturer’s instructions (Alexa Fluor^®^ 488 annexin V/Dead Cell Apoptosis Kit with Alexa^®^ Fluor 488 annexin V and PI for Flow Cytometry, Invitrogen, Carlsbad, CA, USA). Briefly, following OGD injury, the cells were trypsinized without EDTA, harvested, and washed in cold PBS twice. Cells were then treated with annexin V/PI solution in 1 × annexin-binding buffer, and thereafter analyzed using a BD FACS Caliber flow cytometer (BD Biosciences, San Jose, CA). Three separate experiments were performed [26].

### Lactate Dehydrogenase Assay and Enzyme-linked Immunoassay

Cell death was evaluated using the Lactate Dehydragenase (LDH) Cytotoxicity Assay Kit (Beyotime Institute of Biotechnology, Jiangsu, China). SH-SY5Y cells were grown in 96-well plates. After OGD treatment for 6h, the level of LDH in the cell culture supernatant was measured according to the manufacturer’s instruction.

ELISA was used to assess the effects of MLIF on TNF-α and IL-1β levels in OGD-induced BV-2 cells. Cells were plated onto 12-well plate. Supernatants were harvested to measure IL-1β and TNF-α using ELISA kits according to the manufacturer’s instruction.

### Pull-down assay

Cells were cultured until 80% confluent and then used for the pull-down assay. The cells were lysed with M-PER Protein Extraction Reagent (Pierce, Rockford, IL) supplemented with protease inhibitor cocktail in an ice-bath. The protein supernatant was incubated with biotinylated MLIF (40 μL, 5 mg/ml, bio-MLIF) or control solution and incubated for 6−7 h at 4°C. Subsequently, 40 μL of streptavidin-agarose bead suspension (Invitrogen, Carlsbad, CA, USA) was added and stirred gently at 4°C for 4−6 h. The sample was then centrifuged in a microcentrifuge at 12, 000 rpm for 2 min to obtain a pellet (S1), the supernatant was repeated the binding assay for two times to obtain sample2 (S2) and sample3 (S3). After three washes with cold PBS, 1× SDS sample buffer was added to the pellet and boiled. The control groups were respectively showed as C1, C2, and C3. The supernatant was subjected to SDS-PAGE followed by Coomassie Brilliant Blue staining. Proteins were analyzed using matrix-assisted laser desorption/ionization time-of-flight mass spectrometry (Voyager DESTR mass spectrometer, Applied Biosystems, Waltham, MA, USA) after in-gel digestion, and protein identification was performed using the MASCOT search engine (Matrix Science, Boston, MA, USA) [5, 27].

### Confocal microscopy

Cells were seeded onto coverslips and fixed with 4% paraformaldehyde for 15 min, permeabilized with 0.1% Triton-X 100 (Amresco, Solon, OH, USA) for 30 min at room temperature. The cells were blocked with goat serum (Solarbio, Beijing, China) for 1 h at room temperature and stained with eEF1A2 primary antibody overnight at 4°C, and then stained with secondary antibody labeled with phycoerythrin (PE) for 1 h in the dark at room temperature, followed by washing with PBS. MLIF labeled with fluorescein isothiocyanate (FITC-MLIF) and DAPI were separately added to the slides. Stained cells were observed with a Leica TCS SP5 confocal microscope. The images were acquired using LAS-AF software (Leica, Buffalo Grove, IL).

### Western blot analysis

The protein extraction protocol was the same as for the pull-down assay. Briefly, protein concentration was determined using a BCA protein assay kit (Thermo Scientific, Waltham, MA, USA). Equal amounts of total cellular protein were resolved by SDS-PAGE, and subsequently electrotransferred onto a nitrocellulose membrane. After blocking with 5% BSA in TBST (20 mM Tris-HCl, 137 mM NaCl and 0.1% Tween-20, pH 7.4) for 2 h at room temperature, the membranes were incubated with rabbit monoclonal antibody against eEF1A2, p53, caspase3, caspase9, ERK, p-ERK, p38, p-p38, JNK or p-JNK (1:1000) diluted in TBST overnight at 4°C. The membranes were probed with GAPDH antibody as a loading control. The blots were then incubated with the corresponding horseradish peroxidase-conjugated secondary antibody for 1h at room temperature. Immunoreactive proteins were detected using ECL western blotting substrate (Thermo Scientific, Waltham, MA, USA).

### RNA interference

The eEF1A2 siRNA sequences used in this study are 5ʹ-GCGGACCAUCGAGAAGUUUTT-3ʹ (sense) and 5ʹ-AAACUUCUCGAUGGUCCGCTT-3ʹ (antisense). A scrambled siRNA was used as negative control, purchased from GenePharma (Shanghai, China). Transfections were performed using Lipofectamine 2000 reagent (Thermo Scientific, Waltham, MA, USA) according to the manufacturer’s instructions. Cells were used for experiments 48 h after siRNA treatment.

### Hoechst 33258 staining

Cells were rinsed gently three times with ice-cold PBS and fixed in 4% paraformaldehyde for 15 min. After rinsing with PBS for 3 min, cells were stained with 5μg/ml Hoechst 33258 for 15 min. Subsequently, after washing with PBS for 3 min, apoptotic cells were viewed with a Leica SP5 fluorescence microscope with a UV excitation wavelength of 300–500 nm. The images were analyzed using LAS-AF software (Leica, Buffalo Grove, IL). Five different fields were analyzed, and the apoptosis rate was calculated as the ratio of the number of apoptotic cells to the total number of cells (expressed as a percentage) [28].

### Statistical analysis

All data are presented as means ± SEM of three independent experiments. Statistical significance was determined using one-way ANOVA. *P*-values less than 0.05 were considered to indicate statistical significance.

## Results

### MLIF increases cell survival and inhibits apoptosis/necrosis in neuronal ischemic injury

**Fig 1:**
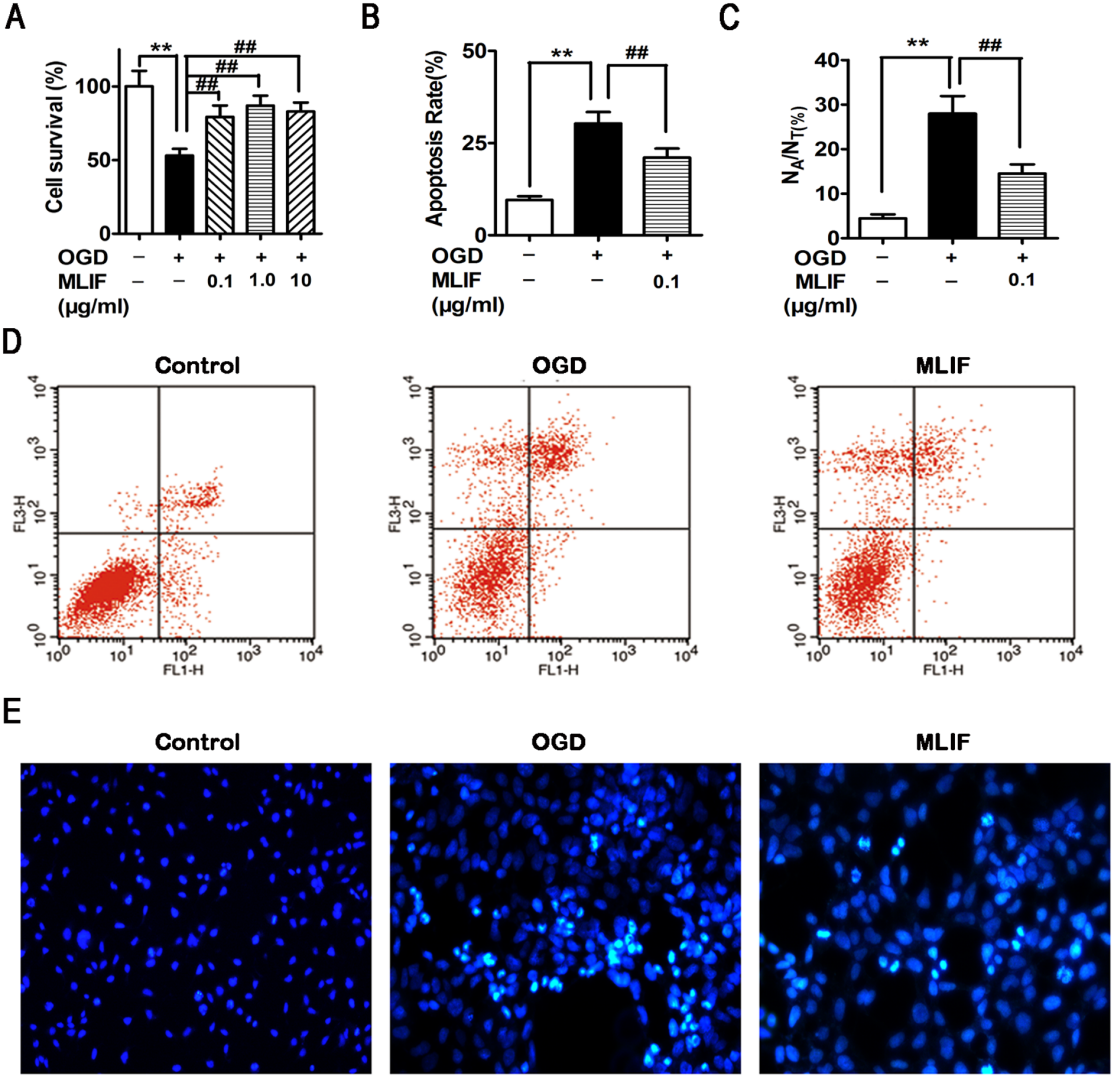
The effect of MLIF on OGD-induced SH-SY5Y neuroblastoma injury was evaluated using MTT assay, flow cytometry and Hoechst staining assay. SH-SY5Y cells were exposed to OGD for 6h. After treatment with MLIF (0.1, 1.0, 10 μg/ml), MTT assay was used to measure the cell survival ratio (A). Annexin V/PI labeling, assessed by flow cytometry (B, D), and Hoechst 33258 staining (C, E) were performed to evaluate apoptosis in SH-SY5Y cells. Data are expressed as the mean ± SEM. Results were analyzed with one-way ANOVA; n = 3. **P < 0.01, OGD group *vs*. control group; ^##^*P*< 0.01, MLIF group *vs*.OGD group.

As shown in Fig 1A, MTT assay revealed that SH-SY5Y cell survival was substantially increased by administration of MLIF (0.1, 1.0, 10 μg/ml), compared with the OGD group. We also detected the effect of MLIF on the survival of primary neurons after OGD and found MLIF improved cell survival (S1 Fig). The number of apoptotic cells was quantitated by flow cytometry assay. The number of apoptotic cells was significantly reduced by treatment with MLIF (0.1 μg/ml) (Fig 1B and 1D). Hoechst 33258 staining assay showed apoptotic cells with condensed or fragmented nuclei and bright blue fluorescence (Fig 1C and 1E). These results indicate that MLIF significantly decreases apoptosis induced by OGD in SH-SY5Y cells. LDH assay was used to investigate the effect of MLIF on the cell necrosis induced by OGD. As shown in S2 Fig, OGD treatment increased LDH release and MLIF (0.1, 1.0μg/ml) significantly decreased LDH release.

In the OGD/R model, the SH-SY5Y cell viability was increased markedly and apoptosis rate was significantly decreaded after treatment with MLIF (0.1μg/ml). MLIF presented the similar neuroprrotective effect on the OGD/R model (S3 Fig).

### MLIF inhibits the expression of apoptosis related protein

**Fig 2:**
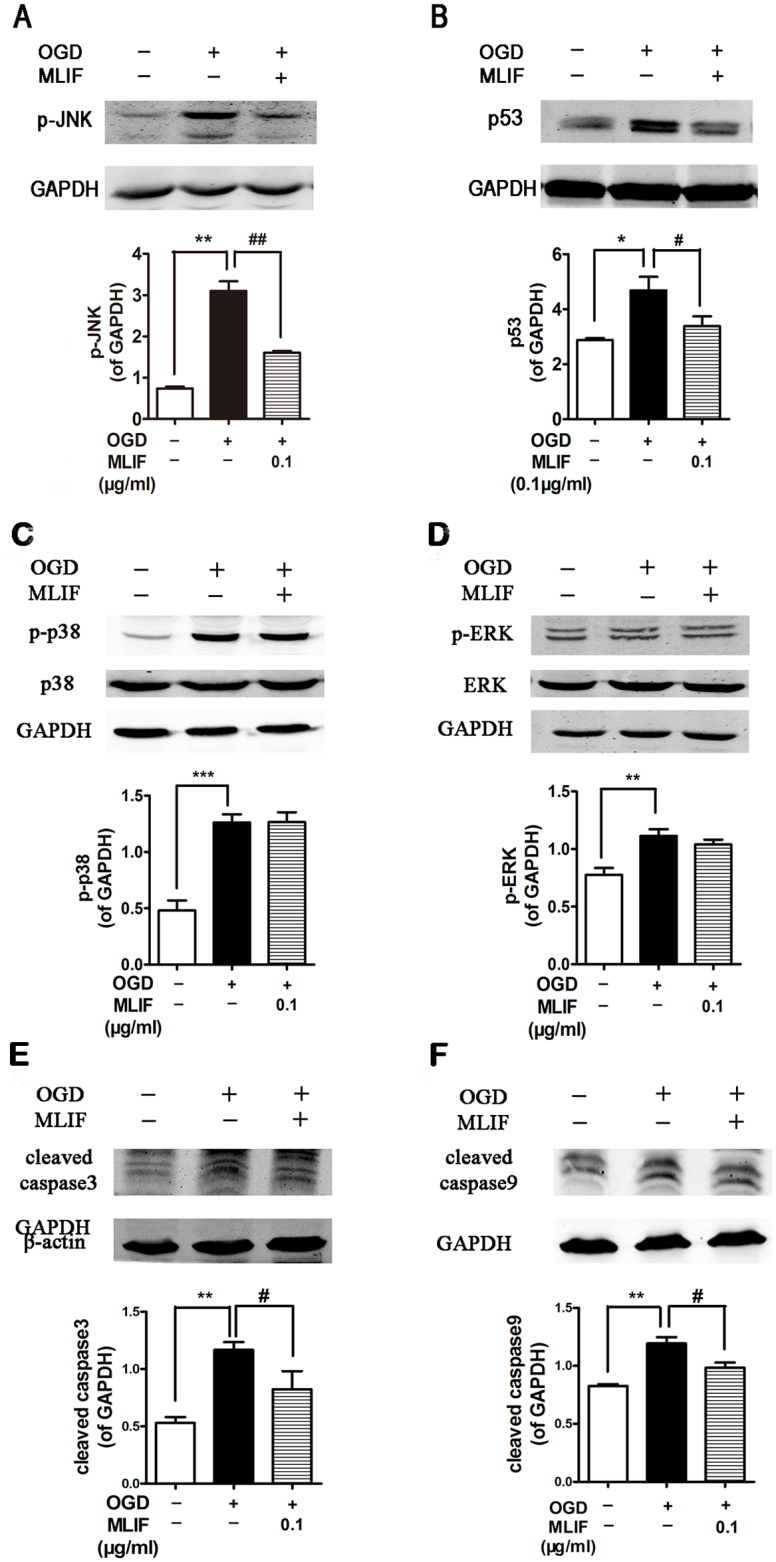
The effect of MLIF on the protein levels of apoptotic proteins in SH-SY5Y cells exposed to OGD. SH-SY5Y cells were exposed to OGD for 6 h with or without incubation with MLIF (0.10 μg/mL). p-JNK (A),p53 (B), p38 (C), ERK (D), caspase3 (E) and caspase9 (F) levels were determined by immunoblotting. Data are expressed as the mean ± SEM. Results were analyzed using one-way ANOVA; n = 3. *P < 0.05, **P < 0.01 or ***P < 0.001, OGD group *vs*. control group; ^#^*P* < 0.05 or ^##^*P* < 0.01 MLIF group *vs*.OGD group.

We evaluated the effect of MLIF on the expression of the apoptosis-related proteins p-JNK, p53, caspase3 and caspase9 in the SH-SY5Y neuroblastoma injury model. p-JNK,p53, cleaved caspase3 and cleaved caspase9 levels were significantly increased in the OGD group, whereas they were significantly decreased in the MLIF group, compared with the OGD group (Fig 2A, 2B, 2E and 2F). The protein expression of total JNK was no significantly altered (S4A Fig). JNK is one member of the MAPKs pathway which plays key roles in regulating cell death and survival from the membrane to the nucleus [29,30]. Therefore, we also examined the impact of MLIF on the expression of p38, p-p38, ERK and p-ERK in OGD-induced SH-SY5Y neuroblastoma injury, and found the levels of p-p38 and p-ERK were significantly increased after OGD treatment, but there was no difference between OGD group and MLIF (0.1μg/ml) treatment group (Fig 2C and 2D).

### MLIF attenuates pro-inflammatory cytokine secretion

Secretion of pro-inflammatory cytokines from microglia which is the primary immune effector cells in the CNS, is the major cause of neuronal cell death associated with neuroinflammation in cerebral ischemia [31,32,33]. We dectected the effect of MLIF on the production of pro-inflammation cytokines IL-1β and TNF-α in BV-2 cells after OGD treatment. ELISA studies revealed IL-1β and TNF-α were significantly depressed by MLIF (S5 Fig).

### MLIF binds to eEF1A2 in neuronal cells

**Fig 3:**
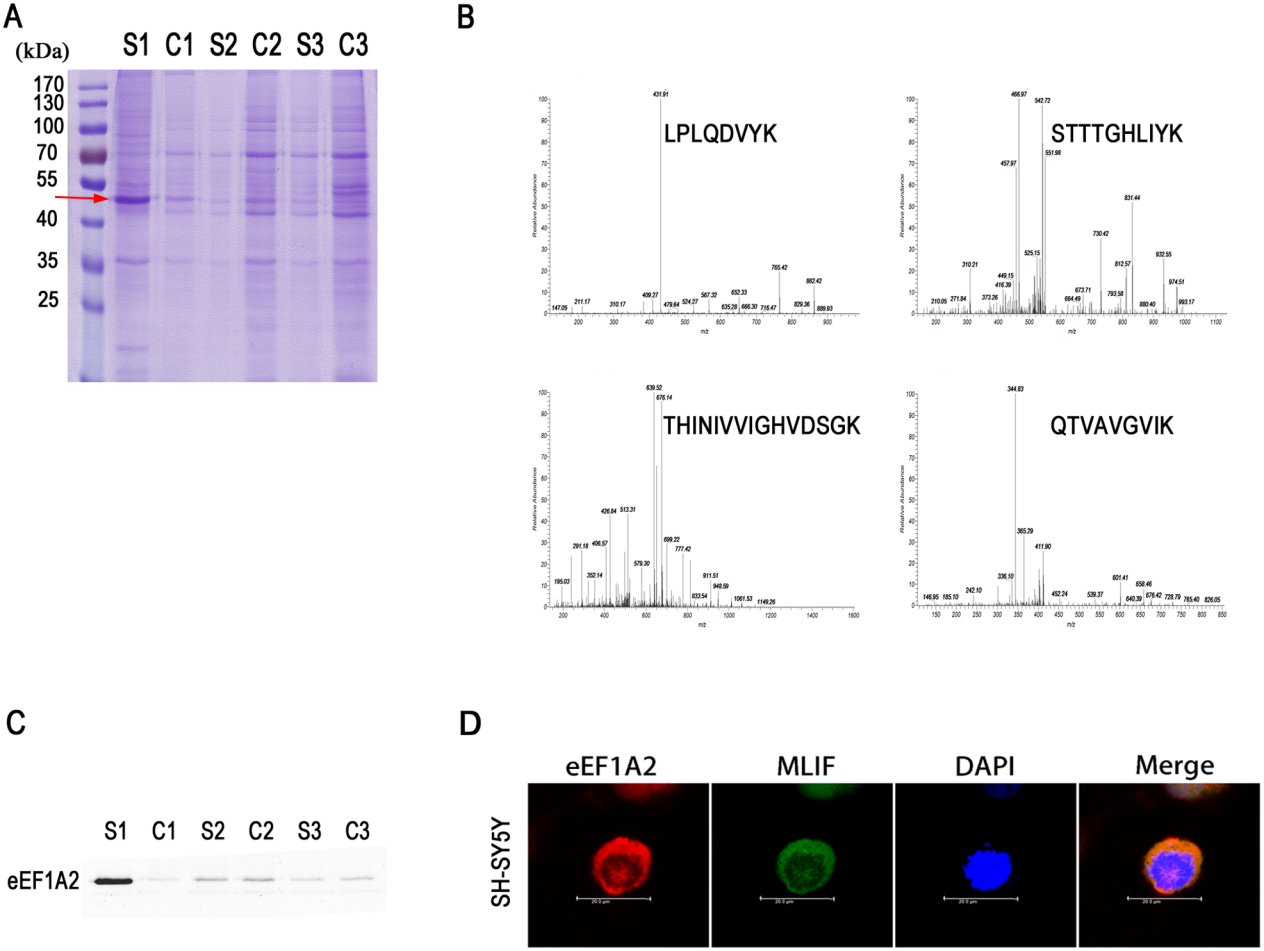
eEF1A2 was identified as the binding protein of MLIF in SH-SY5Y cells. Pull-down assays were carried out using biotin-conjugated MLIF (bio-MLIF) and cell lysates. Binding proteins were washed and separated by SDS-PAGE (A). The binding protein was analyzed by MALDI-TOF MS after in-gel digestion, and found to be eEF1A2 (B). Western blotting with anti-eEF1A2 antibody confirmed the identity of the binding protein (C). Confocal microscopy revealed co-localization of FITC-labeled MLIF (green) and eEF1A2 (labeled with rabbit anti-eEF1A2; red) in SH-SY5Y cells (D).

We used biotin-conjugated MLIF (bio-MLIF) as a bait to identify MLIF binding proteins in SH-SY5Y cells. As shown in Fig 3A, the MLIF binding proteins appeared at ~50 kDa in the S1 lane (red arrow). No significant levels of binding proteins were pulled down in the control group (C lanes). After in-gel digestion, MALDI-TOF mass spectrometry and MASCOT analysis, the target protein was identified as ribosomal protein translation elongation factor eEF1A2 (Fig 3B). We performed western blot analysis on the pull-down samples with an anti-eEF1A2 antibody, which confirmed that the 50 kDa protein was indeed eEF1A2 (Fig 3C). The colocalization of FITC-MLIF and eEF1A2 by confocal microscopy provided further support for the binding interaction between MLIF and eEF1A2 (Fig 3D).

### The neuroprotection provided by MLIF is inhibited by eEF1A2 RNAi in SH-SY5Y cells

**Fig 4:**
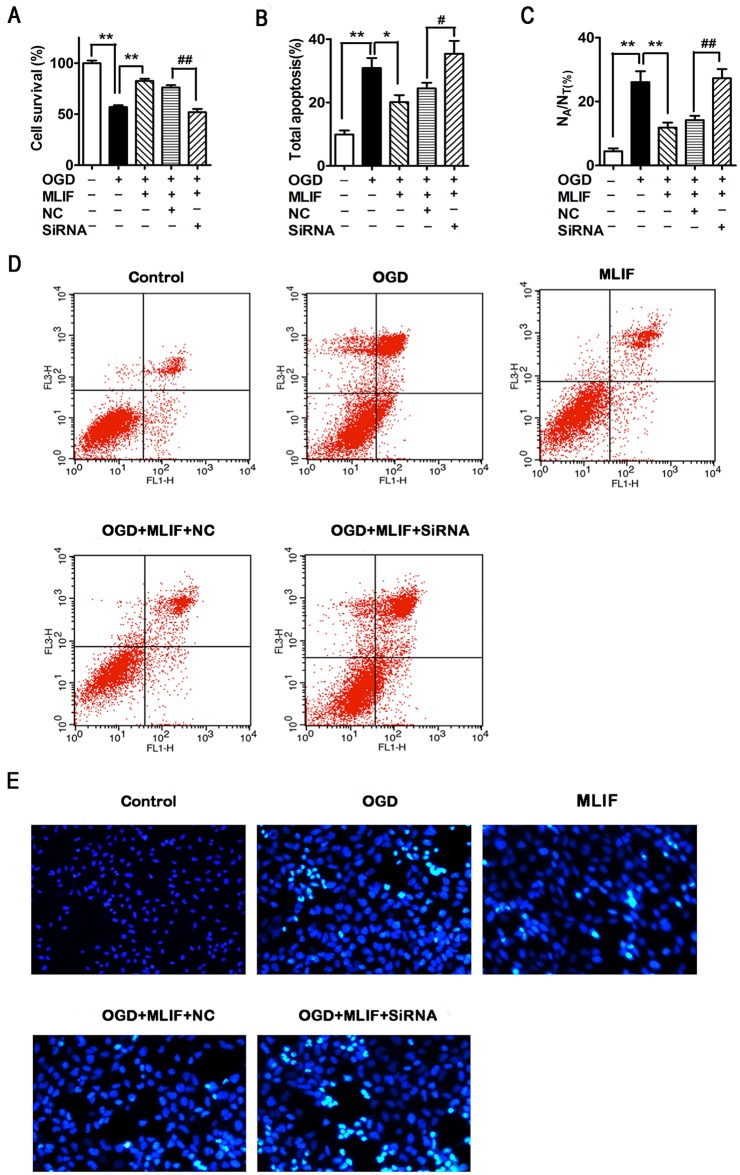
MLIF protects SH-SY5Y cells against apoptosis in an eEF1A2-dependent manner. OGD-exposed SH-SY5Y cells transfected with eEF1A2 siRNA or negative control siRNA (NC) were treated with MLIF (0.1 μg/ml). Cell survival was measured using MTT assay (A). Representative annexin V/PI labeling, assessed by flow cytometry, was used to analyze the ratio of apoptotic SH-SY5Y cells (B, D). Hoechst 33258 staining was used to evaluate the nuclear morphology of SH-SY5Y cells (C, E). Data are expressed as the mean ± SEM. Results were analyzed using one-way ANOVA; n = 3. **P< 0.01 or *P< 0.05, OGD group *vs*. control group or MLIF group; ^##^*P* < 0.01 or ^#^*P*< 0.05, eEF1A2 siRNA group *vs*. NC group.

To examine whether the neuroprotection provided by MLIF was mediated by eEF1A2, we used siRNA to suppress the expression of eEF1A2 in SH-SY5Y cells. MTT assay, annexin V labeling, and propidium iodide and Hoechst 33258 staining were used to evaluate cellular apoptosis[34, 35]. Western blot assay was performed to measure protein expression levels of p-JNK and p53. After transfection with eEF1A2 siRNA, cell survival significantly decreased in the MLIF group compared with the negative control (Fig 4A). Furthermore, the rate of apoptosis was significantly enhanced in eEF1A2 siRNA-treated cells compared with cells treated with the negative control siRNA (Fig 4B and 4D). Hoechst 33258 staining confirmed the results of the MTT and flow cytometry assays (Fig 4C and 4E).

**Fig 5:**
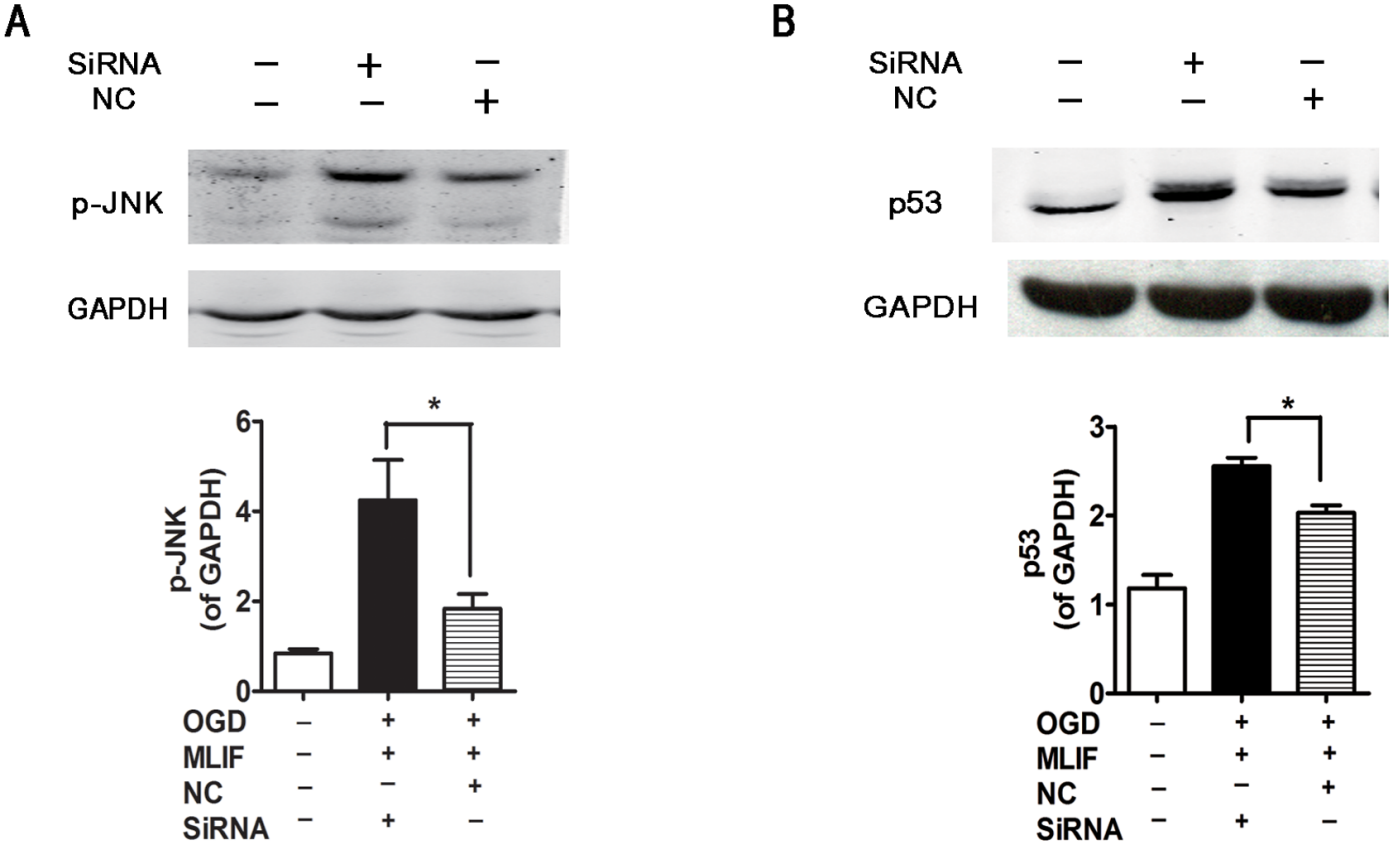
MLIF downregulates p-JNK and p53 levels via eEF1A2. After transfection with eEF1A2 siRNA or NC, OGD-exposed SH-SY5Y cells were incubated with MLIF (0.1 μg/mL). The levels of p-JNK (A) and p53 (B) were determined by immunoblotting. Data are expressed as the mean ± SEM. Results were analyzed using one-way ANOVA; n = 3. *P< 0.01 siRNA group *vs*. NC group.

In cells subjected to eEF1A2 knockdown using siRNA, p-JNK and p53 expression was determined by western blotting. As shown in Fig 5, the inhibitory effect of MLIF on p-JNK and p53 expression was blocked by eEF1A2 siRNA and the protein expression of total JNK was no significantly altered (S4B Fig) indicating that eEF1A2 is essential for the neuroprotective function of MLIF in neural injury.

## Discussion

MLIF, an anti-inflammatory oligopeptide produced by *Entamoeba histolytica* has been reported to have anti-inflammatory effects *in vivo* and *in vitro*. MLIF downregulates iNOS gene expression, increases levels of the anti-inflammatory cytokines IL-10 and TGF-β after spinal cord injury, and inhibits the locomotion of human macrophages and monocytes [36]. In our previous study, we found that MLIF alleviates damage to cerebrovascular endothelial cells induced by hypoxia by inhibiting the expression of adhesion molecules and by targeting the eEF1A1/eNOS pathway [5]. Furthermore, MLIF treatment significantly alleviated neurological deficits and enhanced functional recovery in rats with ischemic or traumatic injury to the spinal cord. However, the mechanisms underlying the neuroprotective effects of MLIF remained unclear.

Nervous system diseases vary in clinical phenotype, and the underlying pathogenetic processes are complex. Neuroprotection often involves cellular defense mechanisms that protect neurons from apoptosis or degeneration [37]. Mitogen-activated protein kinases (MAPKs) are serine/threonine protein kinases including three members: JNK, p38 and ERK, which become phosphorylated and activated in response to external stimulation and regulate cell differentiation, cell survival and apoptosis in neuronal cells [29,30,38]. In the present study, we found that MLIF protects human neuroblastoma SH-SY5Y cells from OGD-induced apoptosis and necrosis through inhibiting caspase-dependent apoptotic protein p-JNK/p53. We subsequently set about to identify MLIF target proteins with a pull-down assay and mass spectrum analysis. Interestingly, we found the target protein was eEF1A2, the main isoform of eEF1A expressed in the mature brain. The importance of eEF1A2 in mediating the effects of MLIF was confirmed by knockdown in SH-SY5Y cells.

There are reports showing that eEF1A2 is involved in Akt-dependent cell migration and actin remodeling [39], apoptosis [40,41], and phosphatidylinositol signaling [42]. eEF1A2 is thought to play a critical role in cell transformation as well. Furthermore, accumulating data show that eEF1A2 has a significant association with nervous system diseases. Deletion of eEF1A2 in mice gives rise to a neurodegenerative phenotype, and the animals die of muscle wasting and neurodegeneration 4 weeks after birth [18]. In addition, eEF1A2 mutation in patients is associated with neurological symptoms and the following clinical features: severe intellectual disability, facial deformity, psychomotor developmental delay, autism and epilepsy [43,44].

Our present findings suggest that eEF1A2 may be a potential new therapeutic target for nervous system diseases. The neuroprotective mechanisms of MLIF targeting eEF1A2 may partial through inhibiting apoptosis. Inactivation of eEF1A proteins leads to immunodeficiency, neural and muscular defects, and enhanced apoptosis [45]. eEF1A2 has a strong connection with apoptosis. Indeed, we observed that the neuroprotective effect of MLIF was mediated by eEF1A2, and was associated with a reduction in apoptosis and reduced expression of the pro-apoptotic proteins p-JNK and p53.

Previous investigations have reported that MAPK pathway is involved in the biological actions of MLIF[46]. As a group of MAPK families, JNK pathway plays an important role in apoptosis by destabilize p53 and promote ubiquitin-mediated degradation [47]. In this work, we found MLIF significantly decreased the expression of p-JNK, and activated caspase-3. MLIF protects OGD-induced SH-SY5Y neuroblastoma injury may through eEF1A2/JNK/P53 pathway. The inhibition of neuronal apoptosis is a key feature of many neuroprotective mechanisms. The c-Jun N-terminal kinases (JNKs), a group of stress kinases activated by cellular stress, have a critical role in apoptosis and play a key role in the nervous system, not only in regulating neuronal death, but also in brain morphogenesis and axodendritic architectonics during development [48,49]. Moreover, under phorbol 12-myristate 13-acetate (PMA) or UV irradiation stress, polysome-associated JNK phosphorylates eEF1A2, thereby increasing levels of polysomal eEF1A2, which in turn induces JNK dissociation in puromycin-treated mammalian cells. Indeed, eEF1A2 and JNK are heavily implicated in cell proliferation and apoptosis [50]. p53, a tumor suppressor, plays a critical role in ischemic injury. When upregulated, p53 induces apoptosis [51, 52]. It is reported that JNK is a regulator of the translation and stability of the p53 protein [53]. Inhibition of eEF1A2 upregulates the levels of the pro-apoptotic proteins p-JNK and p53 in neurons, suggesting that the eEF1A2/JNK/p53 pathway plays a critical role in the neuroprotective action of MLIF. Consequently, eEF1A2 may be a promising new therapeutic target for ischemic brain injury

## Supporting Information

S1 Fig The effect of MLIF on the survival of primary neurons after OGD for 6h using MTT assay. Primary neurons were exposed to OGD for 6h. After treatment with MLIF (0.1, 1.0 μg/ml) at the beginning of OGD, MTT assay was used to measure the cell survival. Data were expressed as the mean ± SEM. Results were analyzed with one-way ANOVA; n = 3. ***P < 0.001, OGD group *vs*. control group; ^###^*P*< 0.001, MLIF group *vs*.OGD group.
(TIF)

S2 Fig The effect of MLIF on OGD-induced SH-SY5Y neuroblastoma injury was evaluated using LDH assay. SH-SY5Y cells were exposed to OGD for 6h. After treatment with MLIF (0.1, 1.0 μg/ml), LDH assay was used to examine necrosis in SH-SY5Y cells. Data were expressed as the mean ± SEM. Results were analyzed with one-way ANOVA; n = 3. ***P < 0.001, OGD group *vs*. control group; ^##^P< 0.01, MLIF group *vs*.OGD group.
(TIF)

S3 Fig The effect of MLIF on OGD/R-induced SH-SY5Y neuroblastoma injury was evaluated using MTT assay and flow cytometry assay. SH-SY5Y cells were exposed to OGD for 4h and reoxygenation for 12h. After treatment with MLIF (0.1, 1.0μg/ml), MTT assay (A) and flowcytometry assay (B,C) were performed to evaluate the effect of MLIF on the cell viability and apoptosis rates in OGD/R-induced cell injury in SH-SY5Y cells. Data were expressed as the mean ± SEM. Results were analyzed with one-way ANOVA; n = 3. **P < 0.01 or ***P < 0.001, OGD/R group vs. control group; ^##^P< 0.01, OGD/R+MLIF group *vs*. OGD/R group.
(TIF)

S4 Fig The effect of MLIF on the total JNK level in SH-SY5Y cells exposed to OGD. A. SH-SY5Y cells were exposed to OGD for 6 h with or without incubation with MLIF (0.1 μg/mL). Total JNK level was determined by immunoblotting. B. After transfection with eEF1A2 siRNA or NC, OGD-exposed SH-SY5Y cells were incubated with MLIF (0.1μg/mL). The levels of total JNK was determined by immunoblotting. Data were expressed as the mean ± SEM. Results were analyzed with one-way ANOVA; n = 3.
(TIF)

S5 Fig MLIF decreased the secretion of inflammatory cytokine IL-1β and TNF-α by BV-2 cells after OGD treatment. After MLIF incubation and OGD treatment for 6 h, mean concentrations of IL-1β (A) and TNF-α (B) in the culture medium of BV-2 cells were examined by ELISA. Data were expressed as the mean ± SEM. Results were analyzed using one-way ANOVA; n = 3. **P < 0.01, OGD group *vs*. control group; ^##^*P* < 0.01 MLIF group *vs*.OGD group.
(TIF)
