# Supplementary figures and images for: MLIF Alleviates SH-SY5Y Neuroblastoma Injury Induced by Oxygen-Glucose Deprivation by Targeting Eukaryotic Translation Elongation Factor 1A2
Source: PLoS One. 2016 Feb 26;11(2):e0149965. doi: 10.1371/journal.pone.0149965 (PMC4769291; doi:10.1371/journal.pone.0149965)

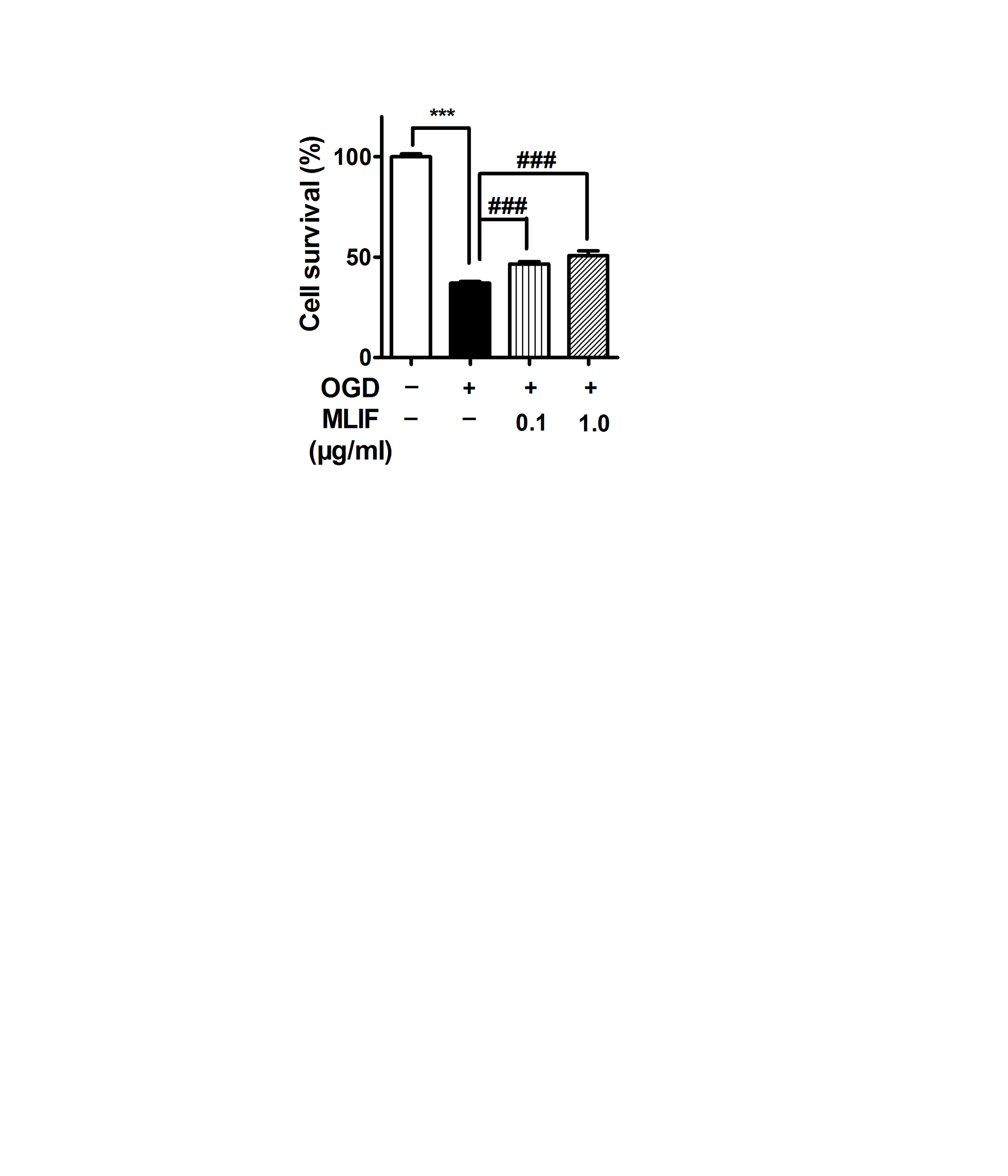

Supplement: S1 Fig — Primary neurons were exposed to OGD for 6h. After treatment with MLIF (0.1, 1.0 μg/ml) at the beginning of OGD, MTT assay was used to measure the cell survival. Data were expressed as the mean ± SEM. Results were analyzed with one-way ANOVA; n = 3. ***P < 0.001, OGD group vs. control group; ###P< 0.001, MLIF group vs.OGD group. (TIF) [file pone.0149965.s001.tif]

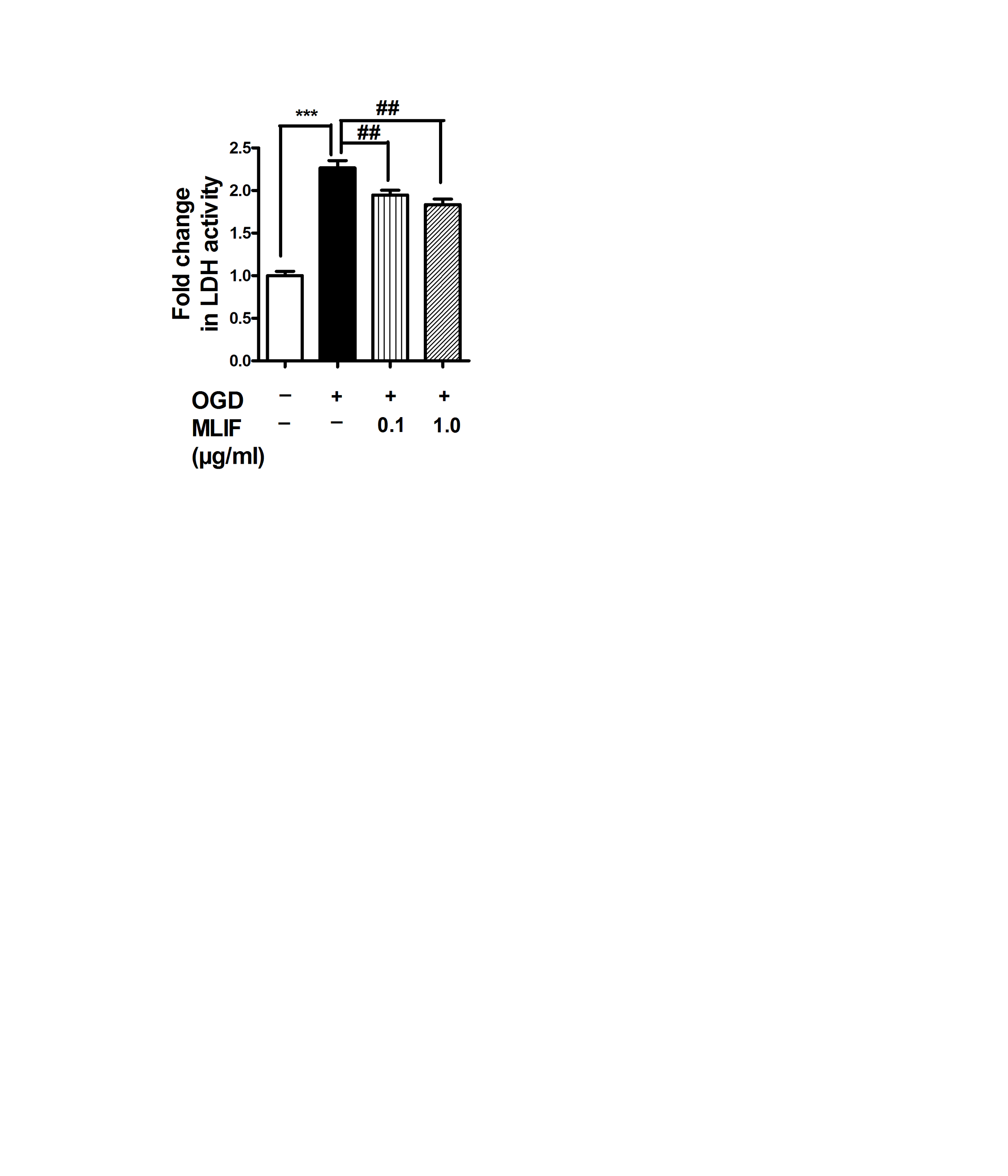

Supplement: S2 Fig — SH-SY5Y cells were exposed to OGD for 6h. After treatment with MLIF (0.1, 1.0 μg/ml), LDH assay was used to examine necrosis in SH-SY5Y cells. Data were expressed as the mean ± SEM. Results were analyzed with one-way ANOVA; n = 3. ***P < 0.001, OGD group vs. control group; ##P< 0.01, MLIF group vs.OGD group. (TIF) [file pone.0149965.s002.tif]

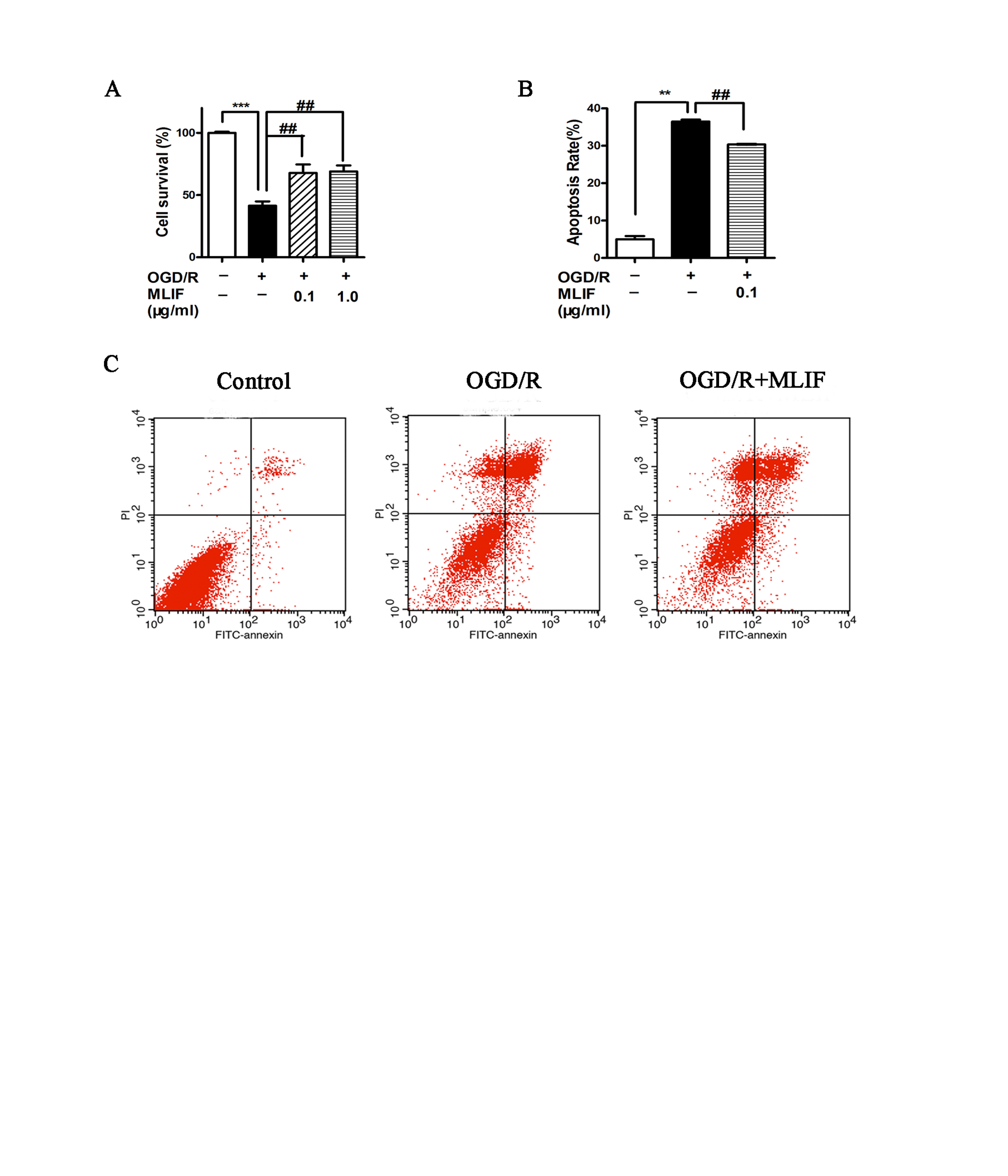

Supplement: S3 Fig — SH-SY5Y cells were exposed to OGD for 4h and reoxygenation for 12h. After treatment with MLIF (0.1, 1.0μg/ml), MTT assay (A) and flowcytometry assay (B,C) were performed to evaluate the effect of MLIF on the cell viability and apoptosis rates in OGD/R-induced cell injury in SH-SY5Y cells. Data were expressed as the mean ± SEM. Results were analyzed with one-way ANOVA; n = 3. **P < 0.01 or ***P < 0.001, OGD/R group vs. control group; ##P< 0.01, OGD/R+MLIF group vs. OGD/R group. (TIF) [file pone.0149965.s003.tif]

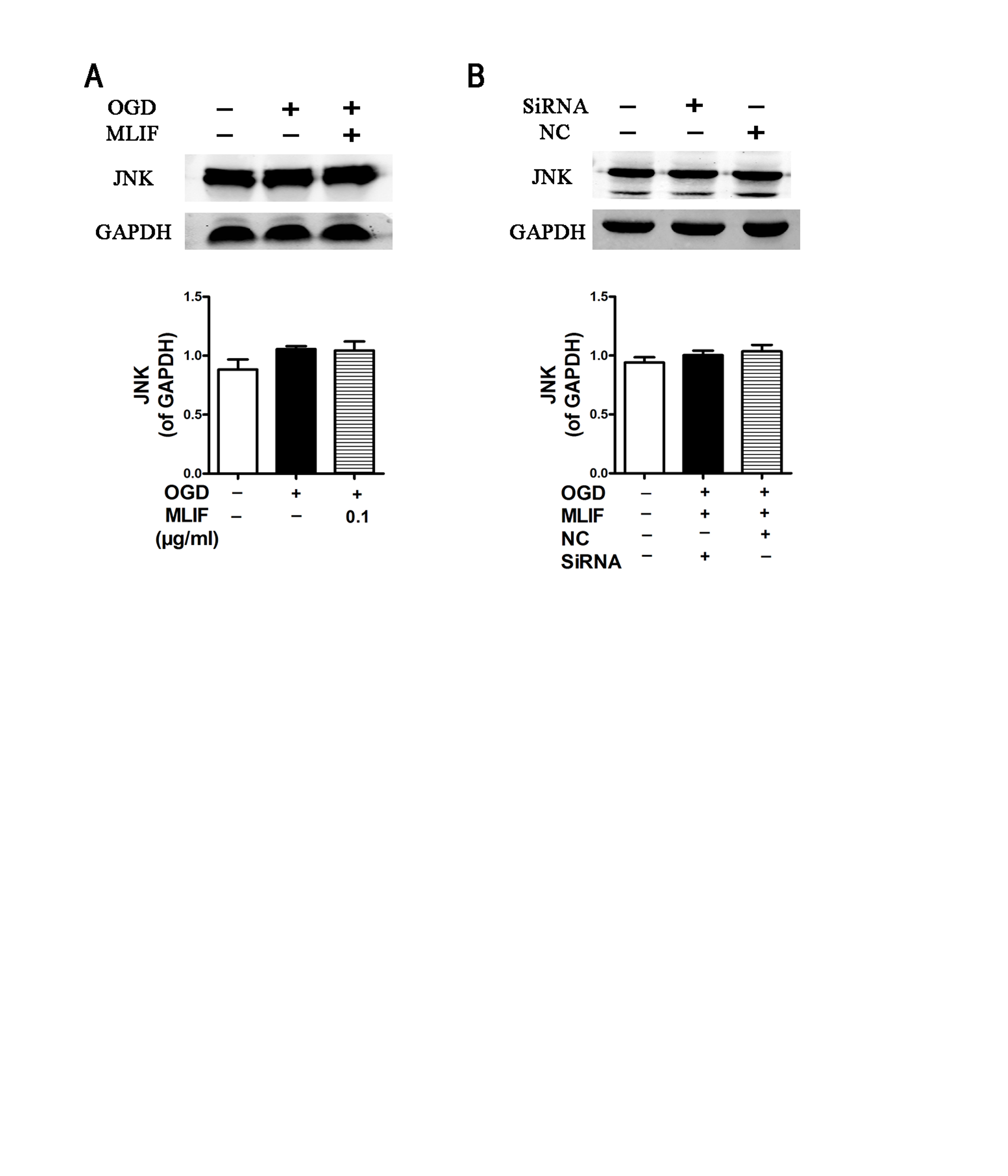

Supplement: S4 Fig — A. SH-SY5Y cells were exposed to OGD for 6 h with or without incubation with MLIF (0.1 μg/mL). Total JNK level was determined by immunoblotting. B. After transfection with eEF1A2 siRNA or NC, OGD-exposed SH-SY5Y cells were incubated with MLIF (0.1μg/mL). The levels of total JNK was determined by immunoblotting. Data were expressed as the mean ± SEM. Results were analyzed with one-way ANOVA; n = 3. (TIF) [file pone.0149965.s004.tif]

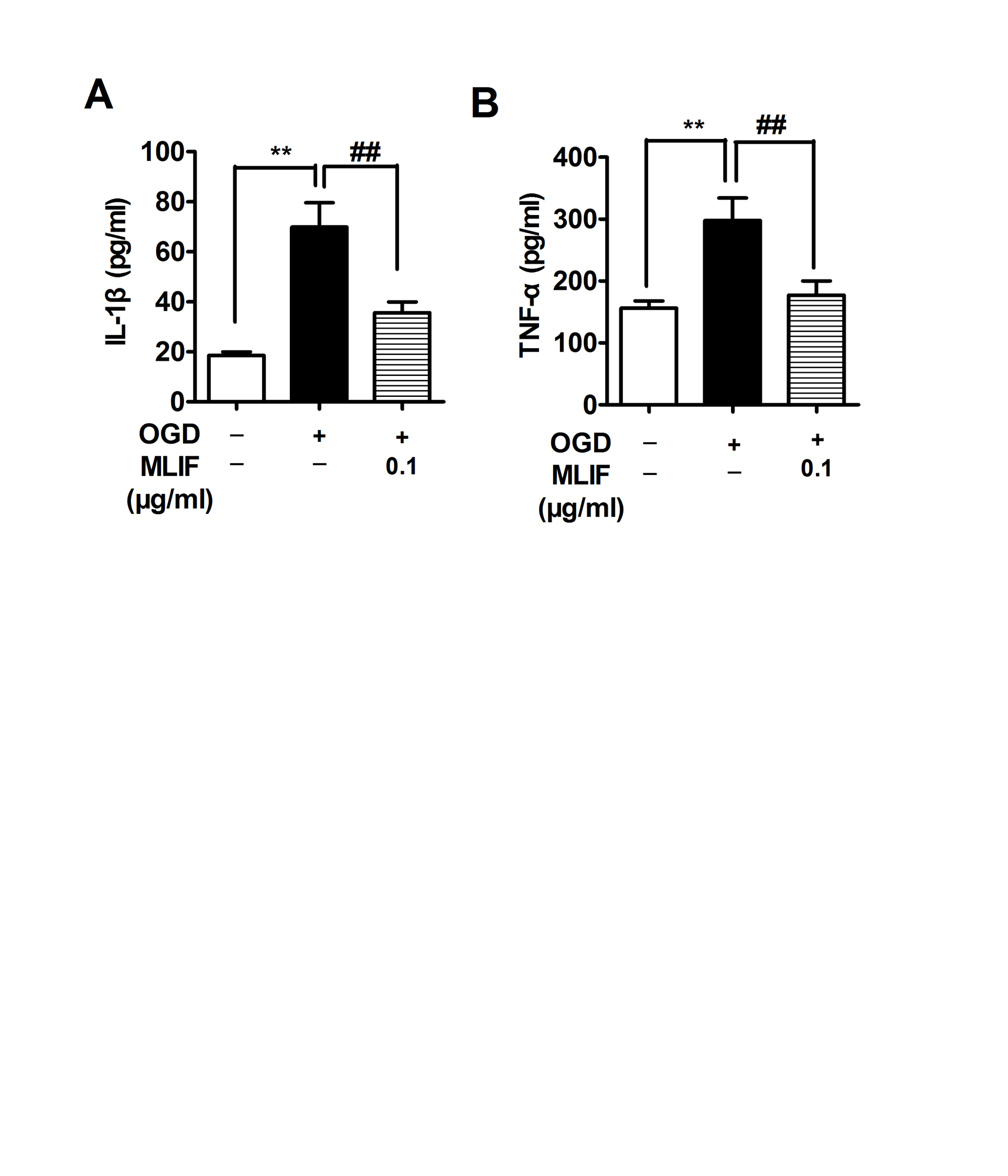

Supplement: S5 Fig — After MLIF incubation and OGD treatment for 6 h, mean concentrations of IL-1β (A) and TNF-α (B) in the culture medium of BV-2 cells were examined by ELISA. Data were expressed as the mean ± SEM. Results were analyzed using one-way ANOVA; n = 3. **P < 0.01, OGD group vs. control group; ##P < 0.01 MLIF group vs.OGD group. (TIF) [file pone.0149965.s005.tif]
